# Low-dose and standard-dose ticagrelor compared with clopidogrel in patients with acute coronary syndromes: A cohort study from china

**DOI:** 10.3389/fcvm.2022.937261

**Published:** 2022-07-26

**Authors:** Wenxing Peng, Yunnan Zhang, Yang Lin

**Affiliations:** ^1^Department of Pharmacy, Beijing Anzhen Hospital, Capital Medical University, Beijing, China; ^2^School of Pharmaceutical Sciences, Capital Medical University, Beijing, China

**Keywords:** ticagrelor, acute coronary syndrome, P2Y12 receptor inhibitor, clopidogrel, platelet function

## Abstract

**Purpose:**

Previous trials have demonstrated that ticagrelor was superior to clopidogrel in acute coronary syndrome (ACS) patients. However, several recent studies showed that ticagrelor was associated with a significantly higher risk of bleeding compared with clopidogrel, especially in East Asian patients. Low-dose ticagrelor might improve the safety of ACS patients in the Chinese population. Therefore, this study mainly explored the low-dose ticagrelor in Chinese ACS patients.

**Methods:**

A total of 199 ACS patients were enrolled in this study. The maximum platelet aggregation rate induced by adenosine-5-diphosphate (ADP) was detected by light transmittance aggregometry (LTA). Platelet aggregation rate induced by ADP of more than or equal to 42.9% was defined as high on-treatment platelet reactivity (HPR) to P2Y12 inhibitors. All patients were followed up for at least 12 months. Clinical outcomes, changes of antiplatelet regimen, medication compliance and adverse reactions were collected.

**Results:**

Patients were divided into three groups according to the P2Y12 inhibitors, including 87 cases in clopidogrel (75 mg once a day) group, 41 cases in ticagrelor 60 mg (twice a day) group, and 71 cases in ticagrelor 90 mg (twice a day) group. ADP-induced platelet aggregation rates in ticagrelor 60 mg group and 90 mg group were 28.4 (19.6, 42.9) and 22.33 (15.1, 34.7) respectively, which were significantly lower than those in clopidogrel group 49.3 (36.5, 61.0) with adjusted *P* < 0.001. At the same time, there was no significant difference in ADP-induced platelet aggregation rate between ticagrelor 60 mg and 90 mg group (adjusted *P* = 0.105). Compared with clopidogrel, the proportion of normal on-treatment platelet reactivity (NPR) of ticagrelor 60 mg and ticagrelor 90 mg were significantly higher than that of clopidogrel, and the proportion of NPR of ticagrelor 90 mg group was significantly higher than that of ticagrelor 60 mg group.

**Conclusions:**

Patients of ticagrelor 60 mg and ticagrelor 90 mg had comparable platelet aggregation rates induced by ADP, and both of them had significantly more potent antiplatelet aggregation activity detected by LTA than clopidogrel.

## Introduction

Acute coronary syndrome (ACS) is a moderate to high fatal cardiovascular disease caused by atherosclerosis, which is one of the leading causes of morbidity and mortality worldwide (1). Dual antiplatelet therapy (DAPT) with aspirin plus P2Y12 inhibitor is the current standard therapy for patients with ACS to prevent major adverse cardiovascular or cerebrovascular events (MACCE) (2, 3).

Clopidogrel, an inactive prodrug, is converted into the active metabolite by two biotransformation steps and its antiplatelet activity is affected by gene polymorphism (4). Thus, clopidogrel is characterized by less potent and variable platelet inhibition. However, ticagrelor directly acts on platelet P2Y12 receptors without enzyme metabolism. When compared with clopidogrel, ticagrelor had a more potent and more predictable antiplatelet aggregation effect. And previous TRITON-TIMI 38 (5) and PLATO trials (6) had demonstrated that ticagrelor and prasugrel were superior to clopidogrel for a primary composite endpoint and that there was no significant difference in the risk of major bleeding between the potent P2Y12 inhibitors and clopidogrel in patients with STEMI and non-ST-elevation ACS. Thus, European and American College of Cardiology (ACC) guidelines suggest that the potent P2Y12 inhibitors ticagrelor and prasugrel are preferred over clopidogrel which only is used when ticagrelor and prasugrel are contraindicated or not available. Recently, several studies showed that ticagrelor was associated with a significantly higher increased the risk of dyspnea, major bleeding and bradycardia events compared with clopidogrel (7–11). And the compliance of patients with ticagrelor had been proved to be lower than that with clopidogrel, which also brought challenges for long-term treatment of ticagrelor (12, 13). Clinical studies also suggested that the de-escalation of antiplatelet therapy in patients with ACS might bring more benefits (14, 15). In addition, compared with Caucasian patients, East Asian patients have a lower rate of ischemic events and a higher rate of bleeding events after PCI, despite a higher on-treatment platelet reactivity, which is referred to as the “East Asian paradox” (16). Therefore, low-dose ticagrelor might improve the safety of ACS patients in the Chinese population. Ticagrelor 60 mg has previously been proved to have comparable efficacy to standard-dose ticagrelor in patients with stable coronary artery disease and have a lower risk of bleeding (17). However, clinical guidelines only recommend ticagrelor 60 mg for patients with ACS after 12 months (18). Thus, there is still a lack of clinical evidence for ACS patients to use low-dose ticagrelor within 12 months. Therefore, this study mainly explored the safety and efficacy of low-dose ticagrelor in Chinese ACS patients.

## Methods

### Study population

ACS patients admitted to the Anzhen Hospital of Capital Medical University from March 2018, to December 2020, were enrolled in the cohort study. Patients were assessed for eligibility for enrollment based on the following inclusion criteria: (1) more than 18 years old; (2) they were diagnosed with ACS, the diagnosis of ACS included unstable angina (UA), non-ST segment elevation myocardial infarction (NSTEMI), and ST-elevation myocardial infarction (STEMI) according to the American Heart Association/American College of Cardiology (AHA/ACC) criteria; (3) patients with life expectancy >1 year. The exclusion criteria were as follows: (1) contraindication to antiplatelet agents (aspirin, clopidogrel or ticagrelor); (2) with active bleeding, bleeding diseases or hematologic disorder; (3) severe hepatic or renal insufficiency (creatinine clearance rate <30 ml/min); (4) total platelet count less than 100 × 10^9^/L; (5) severe anemia (hemoglobin <60 g/L); (6) concomitant administration of other antiplatelet or anticoagulation agents; (7) interruption or change of antiplatelet agents during the follow-up. The study had been approved by the Medical Clinical Research Ethics Committee of Beijing Anzhen Hospital and patient privacy was protected. All patients signed informed consent before they were recruited.

### Platelet function test

The whole blood samples were collected with citrate anticoagulant tubes, and the platelet function was detected by light transmittance aggregometry (LTA). The tubes were preserved and pretreated at laboratory temperature of 25°C within 4 h. The tube was centrifuged at 4,000–5,000 r/min for 5 min to obtain platelet-rich plasma (PRP). Then the remaining plasma that was platelet poor plasma (PPP) was obtained at 22,500 r/min for 15 min. PRP and PPP were used as a reference for establishing the 100% and baseline optical density, respectively. PPP was added to PRP. The platelet concentration was diluted to 2 × 10^5^/μL, and 6 μmol/L adenosine-5-diphosphate (ADP, Rolf Greiner Biochemica, Flacht, Germany) was added to induce platelet aggregation. Then, the platelet aggregation was measured by Chrono-Log LumeAggregometer (model 700, hronoLog, Havertown, Pennsylvania, USA) at instrument temperature of 36.5–37.5°C. The absolute change in optical density was recorded in intervals of 10 min. Aggregation was expressed as the maximum percent change in light transmittance from baseline.

### Definition of high on-treatment platelet reactivity

ADP-induced platelet aggregation rate of more than or equal to 42.9% was defined as high on-treatment platelet reactivity (HPR) to P2Y12 inhibitors. The definition of the cut-off value was based on the results of the previous study (19). The primary endpoint occurred more frequently in patients with ADP-induced platelet aggregation rate ≥ 42.9% at 1-year follow-up than that with ADP-induced platelet aggregation rate <42.9%.

### Follow-up

All patients were followed up for at least 12 months. Clinical outcomes, changes of antiplatelet regimen, medication compliance and adverse reactions were collected during the follow-up by telephone interview, WeChat or clinic visit. Clinical outcomes included bleeding events and dyspnea events. Moderate bleeding events were defined as Bleeding Academic Research Consortium (BARC) class 2, and severe bleeding events were defined as BARC class 3 or higher (20). Severe dyspnea events were defined as incapacitating dyspnea, with the inability to perform normal activities (21).

### Quality control

The accuracy and completeness of data were ensured using the following strategies. First, researchers were trained before the launch of the project to guarantee that the processes of data collection and follow-up were in accordance with standard procedures. Second, platelet function tests were performed by other laboratory technicians who were not involved in the study. And they were blinded to the medication and group of patients. Third, baseline information and clinical characteristics were collected from electronic medical records, and clinical events were verified by at least two clinicians who were blinded to the medication and group of patients.

### Statistical analysis

All statistical analyses were performed with SPSS 23.0 and GraphPad Prism 8. The Kolmogorov-Smirnov test was used to assess whether continuous data were normal distribution or not. Continuous data of normal distribution were presented as means ± SD (standard deviation) and were analyzed by the Student's *t*-test. Non-normally distributed continuous variables were presented as medians and quartiles and were analyzed by the Wilcoxon test. Categorical data were presented as count and percentage and were analyzed by the Chi-square test. Logistic regression analysis was used to adjust concomitant variables. Statistical significance was attached to *P* values <0.05 to allow for group comparisons.

## Results

### Patients characteristics

**Figure 1:**
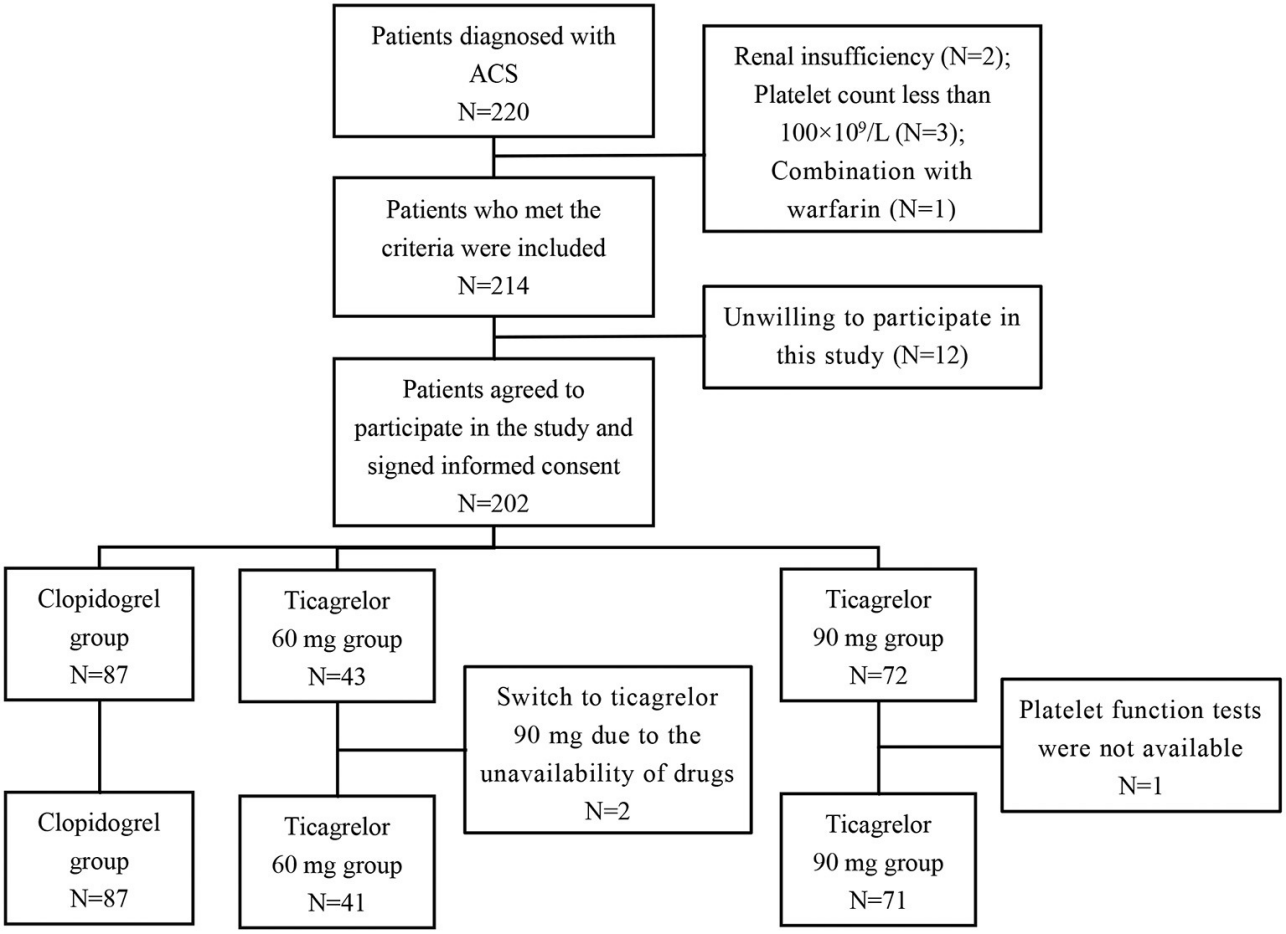
Flowchart of cohort selection. ACS, acute coronary syndrome.

According to the inclusion and exclusion criteria, a total of 199 ACS patients (116 UA, 22 NSTEMI, and 11 STEMI) were enrolled in this study. Figure 1 showed the screening process in this study. The patients were divided into three groups according to the P2Y12 inhibitors, including 87 cases in the clopidogrel (75 mg once a day) group, 41 cases in the ticagrelor 60 mg (twice a day) group, and 71 cases in the ticagrelor 90 mg (twice a day) group. The median follow-up time was 496 (range 389–623) days. Demographic characteristics, cardiovascular risk factors and concomitant medication information of the three groups are shown in Table 1. The mean age of the patients was 59.6 ± 9.7 years, 73.4% were male, 64.3% were hypertensive and 28.1% were diabetic. All patients were treated with aspirin throughout the treatment period. The results showed that there were statistically significant differences in age, the proportion of smokers and ACS type among the three groups. Meanwhile, there were no significant differences among other variables.

**Table 1:** Baseline demographic and clinical characteristics of included patients.

	**Clopidogrel 75 mg**	**Ticagrelor 60 mg**	**Ticagrelor 90 mg**	***P*** **-value**
	***N*** **=** **87**	***N*** **=** **41**	***N*** **=** **71**	
Male sex, *n* (%)	59 (67.8)	28 (68.3)	59 (83.1)	0.069
Age (years)	61.3 ± 9.7	60.2 ± 9.2	57.5 ± 9.7	0.045
Smoker, *n* (%)	22 (25.3)	12 (29.3)	33 (46.5)	0.016
Drinker, *n* (%)	25 (28.7)	7 (17.1)	24 (33.8)	0.163
Body mass index (kg/m^2^)	26.6 ± 3.4	26.2 ± 3	25.9 ± 2.9	0.330
Complication, *n* (%)				
Hypertension	57 (65.5)	28 (68.3)	43 (60.6)	0.679
Diabetes mellitus	21 (24.1)	17 (41.5)	18 (25.4)	0.102
Hyperlipemia	71 (81.6)	29 (70.7)	52 (73.2)	0.296
Prior cerebral infarction	3 (3.4)	1 (2.4)	3 (4.2)	0.884
Prior gastrointestinal ulcer or bleeding	1 (1.1)	0 (0)	1 (1.4)	0.759
Prior cerebral hemorrhage	1 (1.1)	0 (0)	0 (0)	0.524
Prior PCI/CABG	2 (2.3)	3 (7.3)	2 (2.8)	0.328
Laboratory examination				
Platelet count (*10^9^/L)	223.0 (181.5, 249.3)	220.0 (174.5, 247.5)	223.0 (181.5, 249.3)	0.194
Low-density lipoprotein cholesterol (mmol/L)	2.27 (1.84, 2.86)	2.00 (1.64, 2.56)	2.27 (1.84, 2.86)	0.044
Triglycerides (mmol/L)	1.26 (0.94, 1.97)	1.42 (1.04, 2.07)	1.26 (0.94, 1.97)	0.575
Total cholesterol (mmol/L)	4.03 (3.37, 4.72)	3.97 (3.37, 4.49)	4.03 (3.37, 4.72)	0.822
High-density lipoprotein cholesterol (mmol/L)	1 (0.89, 1.25)	1.04 (0.87, 1.21)	1 (0.89, 1.25)	0.748
Serum creatinine (μmol/L)	72.9 ± 18.2	78.7 ± 22.4	75.5 ± 14.1	0.221
UA (μmol/L)	347.6 (300.9, 389.5)	333.8 (277.5, 381.7)	357 (307.5, 407.5)	0.135
Concomitant medication, *n* (%)				
Aspirin	87 (100)	41 (100)	71 (100)	-
ACE inhibitor/ARB	31 (35.6)	10 (24.4)	15 (21.1)	0.109
Beta blocker	20 (23)	16 (39.0)	20 (28.2)	0.170
CCB	17 (19.5)	4 (9.8)	8 (11.3)	0.211
Statin therapy	83 (95.4)	37 (90.2)	68 (95.8)	0.411
PPI	72 (82.8)	27 (65.9)	57 (80.3)	0.085

*PCI, percutaneous coronary intervention; CABG, coronary-artery bypass grafting; UA, uric acid; ACE, angiotensin-converting enzyme; ARB, angiotensin receptor blocker; CCB, calcium channel blocker; PPI, proton pump inhibitor.*

*Continuous data are expressed as means ± SD (standard deviation) or median (1st, 3rd quartiles); Categorical data were presented as count (percentage)*.

### Measures of p2y12 inhibitors response

**Figure 2:**
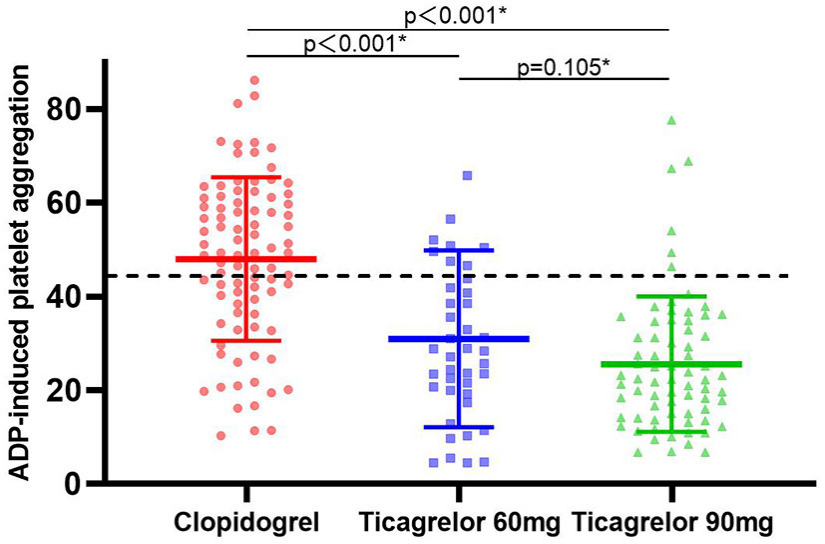
ADP-induced platelet reactivity expressed in clopidogrel, ticagrelor 60 mg and ticagrelor 90 mg groups. The dashed lines indicate threshold values for high platelet reactivity. Solid lines with error bars indicate mean ± SD. ADP, adenosine diphosphate. * Adjusted risk factors of age, smoke by linear regression model.

Platelet function was assessed after at least 1 month of dual antiplatelet therapy in all patients. The maximum platelet aggregation rate induced by ADP in three groups was detected by the LTA method. Figure 2 presented the distribution of ADP-induced platelet aggregation rates among the three groups. ADP-induced platelet aggregation rates in ticagrelor 60 mg group and 90 mg group were 28.4 (19.6, 42.9) and 22.33 (15.1, 34.7), respectively, which were significantly lower than those in clopidogrel group 49.3 (36.5, 61.0) with adjusted *P* < 0.001. At the same time, there was no significant difference in ADP-induced platelet aggregation rate between ticagrelor 60 mg and 90 mg group (adjusted *P* = 0.105).

### High on-treatment platelet reactivity

The maximum platelet aggregation rate induced by ADP ≥ 42.9% was defined as HPR, and the rate <42.9% was considered to achieve the effect of antiplatelet therapy and was defined as normal on-treatment platelet reactivity (NPR). There were 56 (64.4%) patients with HPR in clopidogrel group, 10 (24.4%) patients in ticagrelor 60 mg group and 6 (8.5%) patients in ticagrelor 90 mg group. Compared with clopidogrel, the proportion of NPR of ticagrelor 60 mg and ticagrelor 90 mg were significantly higher than that of clopidogrel (adjusted OR 5.762, 95% CI 2.458–13.508, *P* < 0.001 and adjusted OR 21.204, 95% CI 7.860–57.201, *P* < 0.001), and the proportion of NPR of ticagrelor 90 mg group were significantly higher than that of ticagrelor 60 mg group (adjusted OR 3.623, 95% CI 1.174–11.185, *P* = 0.025) (Table 2).

**Table 2:** Platelet reactivity in patients with ACS.

**Group**	**HPR**	**NPR**	**NPR**	***P*** **value**	**NPR**	**Adjusted** ***P*** value*
	***n*** **(%)**	***n*** **(%)**	**Odds ratio (95%CI)**		Adjusted odds ratio (95%CI)*	
Clopidogrel	56 (64.4)	31 (35.6)	–	–	–	
Ticagrelor 60 mg	10 (24.4)	31 (75.6)	5.600 (2.425, 12.933)^a^	<0.001^a^	5.762 (2.458, 13.508)^a^	<0.001^a^
Ticagrelor 90 mg	6 (8.5)	65 (91.5)	19.570 (7.611, 50.319)^a^	<0.001^a^	21.204 (7.860, 57.201)	<0.001^a^
			3.495 (1.165, 10.486)^b^	0.026^b^	3.623 (1.174, 11.185)^b^	0.025^b^

*HPR, high on-treatment platelet reactivity, platelet reactivity index ≥ 42.9; NPR, normal on-treatment platelet reactivity, platelet reactivity index <42.9.*

*^a^Compared with clopidogrel; ^b^ Compared with ticagrelor 60 mg group.*

*Adjusted risk factors of age, smoke by logistic regression model.

### Adverse events

All patients were followed up for more than 1 year. No death or myocardial infarction occurred during the follow-up period. There was one patient in clopidogrel group who was hospitalized for ischemic stroke 2 months after enrollment and continued dual antiplatelet therapy after discharge. Due to the small sample size and the low incidence of cardiovascular adverse events, this study did not compare the cardiovascular adverse events among the groups (Table 3).

**Table 3:** The occurrence of bleeding and dyspnea events in ACS patients.

**Events**	**Clopidogrel**	**Ticagrelor**	**Ticagrelor**	***P***	**Adjusted** ***P*** value*
	**75 mg**	**60 mg**	**90 mg**		
	**N** **=** **87**	**N** **=** **41**	**N** **=** **71**		
Bleeding events	7 (8.0)	5 (12.2)	12 (16.9)	0.236	0.121
Moderate bleeding events (BARC2)	0 (0)	0 (0)	3 (4.2)	0.064	0.011
Severe bleeding events (BARC ≥ 3)	0 (0)	0 (0)	0 (0)	–	–
Dyspnea events	3 (3.4)	4 (9.8)	12 (16.9)	0.017	0.019
Severe dyspnea events	0 (0)	1 (2.4)	6 (8.5)	0.124	0.130
Treatment interruption due to bleeding or dyspnea events	0 (0)	1 (2.4)	5 (7.0)	0.758	0.613

*ACS, acute coronary syndrome; BARC, Bleeding Academic Research Consortium.*

*Adjusted risk factors of age, smoke by logistic regression model.

## Discussion

This study compared the efficacy and safety of ticagrelor 60 mg, ticagrelor 90 mg and clopidogrel in patients with ACS, and discussed the application of ticagrelor de-escalation strategy in patients with ACS. The main results showed that: (1) ADP-induced platelet aggregation rates of ticagrelor 60 mg and ticagrelor 90 mg were significantly lower than that of clopidogrel 75 mg in patients with ACS. (2) ADP-induced platelet aggregation rate of ticagrelor 60 mg was slightly higher than that of ticagrelor 90 mg, but there was no significant difference.

In this study, the age of patients in ticagrelor 90 mg group was lower than clopidogrel group and ticagrelor 60 mg group. This reason might be that younger patients had a lower risk of bleeding, and clinicians preferred to select the stronger P2Y12 inhibitor. The mean age of the ACS patients in the study was about 60 years, which seemed to be younger than Caucasian populations, but it was similar to that of ACS patients from the previous studies in China (22–24). The main reasons include: (1) China a developing country, many patients do not receive sufficient medical care, such as follow-up visits by family doctors, careful monitoring of blood glucose and blood pressure; (2) Many patients in China are heavy manual workers, which is also an important risk factor for ischemic heart disease (25). At the same time, the proportion of smokers in ticagrelor 90 mg group is higher. Although previous studies showed that these factors had a small effect on the ADP-induced platelet aggregation rate measured by LTA (26), we adjusted for these confounding factors, which had no significant effect on the final results. LTA is a common platelet aggregation test to evaluate antiplatelet function *in vitro*, even if it has some limitations, such as poor repeatability and tedious procedures. And high platelet aggregation rate has been proved to be an independent risk factor for adverse cardiovascular disease (18). Thus, the ADP-induced platelet aggregation rate measured by LTA was applied as an alternative outcome to assess the effectiveness of P2Y12 inhibitors.

The results of our study were basically consistent with previous studies. The STEEL-PCI study, a randomized controlled trial of 180 patients with SCAD after PCI, compared the antiplatelet aggregation effects of ticagrelor 90 mg, ticagrelor 60 mg and clopidogrel 75 mg (17). The results showed that the antiplatelet aggregation activities of ticagrelor 90 mg and ticagrelor 60 mg were better than that of clopidogrel, even in patients who were not carriers of CYP2C19 loss-of-function alleles. PEGASUS-TIMI54 is a large, international multicenter randomized controlled trial involving 21,000 patients with SCAD with previous myocardial infarction (1–3 years) (18). The study compared ticagrelor 90 and 60 mg with placebo, respectively, and confirmed that ticagrelor combined with low-dose aspirin reduced the risk of cardiovascular adverse events (including cardiovascular death, myocardial infarction or stroke). Another study from PEGASUS-TIMI 54 trial compared the platelet inhibition for ticagrelor 60 and 90 mg with placebo, and it focused on the long-term prevention of ischemic events in patients with prior myocardial infarction (27). Although the main results were similar to the previous study, the control group of our study was clopidogrel instead of placebo and we focused on patients with ACS instead of SCAD.

Some previous studies also explored the antiplatelet aggregation activity of low-dose ticagrelor in the Chinese population. Li et al. compared platelet inhibition rates of low-dose ticagrelor 45 mg, standard-dose of ticagrelor 90 mg and clopidogrel in Chinese healthy volunteers (26). The results showed that there was no significant difference in platelet inhibition rate between the low-dose ticagrelor group and the standard-dose ticagrelor group, and both were significantly higher than those in the clopidogrel group.

Prasugrel is not licensed in China, and therefore ticagrelor is the only novel P2Y12 inhibitor. Although ticagrelor is recommended as a first-line P2Y12 inhibitor, clopidogrel remains widely prescribed in China. A study by Li et al. reported that 89.3% of patients undergoing PCI were treated with clopidogrel in China (28). Clopidogrel has been on the market for a long time and it is more economical and accessible in China. Moreover, compared with Caucasian populations, patients from East Asia have a significantly higher risk of bleeding with a lower risk of ischemia (16). Thus, clinicians are more willing to choose clopidogrel to avoid major bleeding. However, some evidence showed that ticagrelor had cardioprotective effects against acute myocardial ischemia/reperfusion injury, reduced infarct size and the excessive inflammatory response to myocardial infarction beyond its inhibitory effects on platelet aggregation (29–31). Thus, optimizing long-term treatment with ticagrelor might bring more benefits to patients with ACS.

Improving the efficacy and safety of dual antiplatelet therapy in patients with ACS is the focus of optimizing the secondary prevention of cardiovascular disease. And the antiplatelet drug de-escalation strategy has gradually attracted increasing attention. TOPIC trial (32) and TWILIGHT trial (33) also explored the efficacy and safety of antiplatelet drug de-escalation therapy for patients after PCI. Although the therapy and time of de-escalation were different, these researches all suggested that antiplatelet de-escalation therapy might reduce the risk of bleeding events. Low-dose ticagrelor might be an alternative for antiplatelet therapy in patients with ACS. Ticagrelor 60 mg for ACS patients within 12 months is still off-label drug use in China, but it has been gradually used by clinicians to reduce the incidence of adverse events. Bleeding events and dyspnea events are the major limitations of the potent antiplatelet agent ticagrelor. In our study, the incidence of bleeding events was the highest in ticagrelor 90 mg and slightly lower in ticagrelor 60 mg. Similarly, the incidence of dyspnea in ticagrelor 60 mg was lower and the incidence of severe dyspnea and treatment interruption events decreased compared with that in ticagrelor 90 mg. Thus, the main results from this study provided some evidence to support ticagrelor 60 mg for ACS patients.

Due to the low incidence of adverse events, we did not further compare whether there was a significant difference in the incidence of events among the groups. STEEL-PCI trial (17) also showed that the rates of bleeding and dyspnea were lower with the ticagrelor 60 mg dose than with the ticagrelor 90 mg dose. In addition, a phase III, randomized, multicenter study is currently being conducted to investigate the efficacy of low-dose ticagrelor treatment, which focus on de-escalation antiplatelet strategies after 30 days following ACS (34). More evidence of low-dose ticagrelor is expected in the future.

This study also confirmed that there was no significant difference in antiplatelet aggregation rate after ticagrelor 90 mg de-escalation to 60 mg maintenance therapy, but the proportion of NPR in ticagrelor 90 mg group was significantly higher than that in ticagrelor 60 mg group. Limited by the sample size of this study, it is difficult to evaluate the clinical benefits of patients with a de-escalation strategy. The effect of 60 mg treatment with ticagrelor is expected, and we will further explore the efficacy and safety of antiplatelet degradation therapy in subsequent studies.

## Limitations

There were still some limitations in this study: (1) This study is a single-center observational cohort study. Thus, there were potential publication bias and selection bias. We adjusted for related confounding factors by logistic regression analysis to balance these biases; (2) Due to the limitation of the small sample size of cases, ischemic cardiovascular events were not the primary endpoint in this study, with an alternative outcome of platelet aggregation rate induced by ADP; (3) Although all patients have been followed up at least 1 year, it is difficult to evaluate the cardiovascular adverse events and safety in patients with three different antiplatelet therapy due to the small sample size. Thus, more large sample studies are still needed to verify the efficacy and safety of low-dose ticagrelor.

In conclusion, the platelet aggregation rates of ticagrelor 60 mg and ticagrelor 90 mg were significantly lower than that of clopidogrel 75 mg in patients with ACS. And there was no significant difference between platelet aggregation rate of ticagrelor 60 mg and ticagrelor 90 mg.

## Data availability statement

The raw data supporting the conclusions of this article will be made available by the authors, without undue reservation.

## Ethics statement

Written informed consent was obtained from the individual(s) for the publication of any potentially identifiable images or data included in this article.

## Author contributions

Material preparation, data collection, and analysis were performed by WP. YL participated in analysis and wrote part of the manuscript. All authors contributed to the study's conception and design. All authors contributed to the article and approved the submitted version.

## Funding

This work was funded by National Major Scientific and Technological Special Project for - Significant New Drugs Development during the Thirteenth Five-year Plan Period (2017ZX09304017).

## Conflict of interest

The authors declare that the research was conducted in the absence of any commercial or financial relationships that could be construed as a potential conflict of interest.

## Publisher's note

All claims expressed in this article are solely those of the authors and do not necessarily represent those of their affiliated organizations, or those of the publisher, the editors and the reviewers. Any product that may be evaluated in this article, or claim that may be made by its manufacturer, is not guaranteed or endorsed by the publisher.

## References

[1] Kamran H Jneid H Kayani WT Virani SS Levine GN Nambi V. Oral antiplatelet therapy after acute coronary syndrome: a review. Jama. (2021) 325:1545–55. 10.1001/jama.2021.0716 33877270

[2] Amsterdam EA Wenger NK Brindis RG Casey DE Jr Ganiats TG Holmes DR Jr. 2014 AHA/ACC guideline for the management of patients with non-ST-elevation acute coronary syndromes: a report of the American College of Cardiology/American Heart Association task force on practice guidelines. J Am Coll Cardiol. (2014) 64:e139–228. 10.1016/j.jacc.2014.09.017 25260718

[3] Collet JP Thiele H Barbato E Barthélémy O Bauersachs J Bhatt DL. 2020 ESC Guidelines for the management of acute coronary syndromes in patients presenting without persistent ST-segment elevation. Eur Heart J. (2021) 42:1289–367. 10.1093/eurheartj/ehaa575 32860058

[4] Mega JL Close SL Wiviott SD Shen L Hockett RD Brandt JT. Cytochrome p-450 polymorphisms and response to clopidogrel. N Engl J Med. (2009) 360:354–62. 10.1056/NEJMoa0809171 19106084

[5] Wiviott SD Braunwald E McCabe CH Montalescot G Ruzyllo W Gottlieb S. Prasugrel versus clopidogrel in patients with acute coronary syndromes. N Engl J Med. (2007) 357:2001–15. 10.1056/NEJMoa0706482 17982182

[6] James S Akerblom A Cannon CP Emanuelsson H Husted S Katus H. Comparison of ticagrelor, the first reversible oral P2Y(12) receptor antagonist, with clopidogrel in patients with acute coronary syndromes: rationale, design, and baseline characteristics of the PLATelet inhibition and patient Outcomes (PLATO) trial. Am Heart J. (2009) 157:599–605. 10.1016/j.ahj.2009.01.003 19332184

[7] Guan W Lu H Yang K. Choosing between ticagrelor and clopidogrel following percutaneous coronary intervention: a systematic review and meta-analysis (2007–2017). Medicine (Baltimore). (2018) 97:e12978. 10.1097/md.0000000000012978 30412125 PMC6221558

[8] Sun M Cui W Li L. Comparison of clinical outcomes between ticagrelor and clopidogrel in acute coronary syndrome: a comprehensive meta-analysis. Front Cardiovasc Med. (2021) 8:818215. 10.3389/fcvm.2021.818215 35155618 PMC8829718

[9] Chen IC Lee CH Fang CC Chao TH Cheng CL Chen Y. Efficacy and safety of ticagrelor versus clopidogrel in acute coronary syndrome in Taiwan: a multicenter retrospective pilot study. J Chin Med Assoc. (2016) 79:521–30. 10.1016/j.jcma.2016.02.010 27339180

[10] Misumida N Aoi S Kim SM Ziada KM Abdel-Latif A. Ticagrelor versus clopidogrel in East Asian patients with acute coronary syndrome: Systematic review and meta-analysis. Cardiovasc Revasc Med. (2018) 19:689–94. 10.1016/j.carrev.2018.01.009 29452843

[11] Wu B Lin H Tobe RG Zhang L He B. Ticagrelor versus clopidogrel in East-Asian patients with acute coronary syndromes: a meta-analysis of randomized trials. J Comp Eff Res. (2018) 7:281–91. 10.2217/cer-2017-0074 29094604

[12] Dayoub EJ Seigerman M Tuteja S Kobayashi T Kolansky DM Giri J. Trends in platelet adenosine diphosphate P2Y12 receptor inhibitor use and adherence among antiplatelet-naive patients after percutaneous coronary intervention, 2008–2016. JAMA Intern Med. (2018) 178:943–50. 10.1001/jamainternmed.2018.0783 29799992 PMC6145718

[13] Suh K Basu A Carlson JJ Branch KR. Exploring medication adherence with P2Y(12) inhibitors using conditional and unconditional quantile regression approaches. Am J Cardiovasc Drugs. (2021) 21:193–204. 10.1007/s40256-020-00405-1 32232734

[14] Sibbing D Aradi D Jacobshagen C Gross L Trenk D Geisler T. Guided de-escalation of antiplatelet treatment in patients with acute coronary syndrome undergoing percutaneous coronary intervention (TROPICAL-ACS): a randomised, open-label, multicentre trial. Lancet. (2017) 390:1747–57. 10.1016/s0140-6736(17)32155-4 28855078

[15] Szummer K Montez-Rath ME Alfredsson J Erlinge D Lindahl B Hofmann R. Comparison between ticagrelor and clopidogrel in elderly patients with an acute coronary syndrome: insights from the SWEDEHEART registry. Circulation. (2020) 142:1700–8. 10.1161/circulationaha.120.050645 32867508

[16] Kang J Kim HS. The evolving concept of dual antiplatelet therapy after percutaneous coronary intervention: focus on unique feature of East Asian and “Asian Paradox”. Korean Circ J. (2018) 48:537–51. 10.4070/kcj.2018.0166 29968428 PMC6031716

[17] Orme RC Parker WAE Thomas MR Judge HM Baster K Sumaya W. Study of two dose regimens of ticagrelor compared with clopidogrel in patients undergoing percutaneous coronary intervention for stable coronary artery disease (STEEL-PCI). Circulation. (2018) 138:1290–300. 10.1161/circulationaha.118.034790 29930021 PMC6159686

[18] Bonaca MP Bhatt DL Cohen M Steg PG Storey RF Jensen EC. Long-term use of ticagrelor in patients with prior myocardial infarction. N Engl J Med. (2015) 372:1791–800. 10.1056/NEJMoa1500857 25773268

[19] Breet NJ van Werkum JW Bouman HJ Kelder JC Ruven HJ Bal ET. Comparison of platelet function tests in predicting clinical outcome in patients undergoing coronary stent implantation. JAMA. (2010) 303:754–62. 10.1001/jama.2010.181 20179285

[20] Ndrepepa G Schuster T Hadamitzky M Byrne RA Mehilli J Neumann FJ. Validation of the bleeding academic research consortium definition of bleeding in patients with coronary artery disease undergoing percutaneous coronary intervention. Circulation. (2012) 125:1424–31. 10.1161/circulationaha.111.060871 22344040

[21] Storey RF Bliden KP Patil SB Karunakaran A Ecob R Butler K. Incidence of dyspnea and assessment of cardiac and pulmonary function in patients with stable coronary artery disease receiving ticagrelor, clopidogrel, or placebo in the ONSET/OFFSET study. J Am Coll Cardiol. (2010) 56:185–93. 10.1016/j.jacc.2010.01.062 20620737

[22] Zhang Y Zhang Y Shi X Lin B Han J Wang Y. Clopidogrel versus ticagrelor in CYP2C19 loss-of-function allele noncarriers: a real-world study in China. Thromb Haemost. (2022) 122:842–52. 10.1055/s-0041-1735193 34428831

[23] Zhao Q Zhang TY Cheng YJ Ma Y Xu YK Yang JQ. Impacts of triglyceride-glucose index on prognosis of patients with type 2 diabetes mellitus and non-ST-segment elevation acute coronary syndrome: results from an observational cohort study in China. Cardiovasc Diabetol. (2020) 19:108. 10.1186/s12933-020-01086-5 32641127 PMC7341665

[24] Liu R Lyu SZ Zhao GQ Zheng W Wang X Zhao XD. Comparison of the performance of the CRUSADE, ACUITY-HORIZONS, and ACTION bleeding scores in ACS patients undergoing PCI: insights from a cohort of 4939 patients in China. J Geriatr Cardiol. (2017) 14:93–9. 10.11909/j.issn.1671-5411.2017.02.011 28491083 PMC5409350

[25] Vågerö D Norell SE. Mortality and social class in Sweden–exploring a new epidemiological tool. Scand J Soc Med. (1989) 17:49–58. 10.1177/140349488901700109 2711146

[26] Li P Gu Y Yang Y Chen L Liu J Gao L. Low-dose ticagrelor yields an antiplatelet efficacy similar to that of standard-dose ticagrelor in healthy subjects: an open-label randomized controlled trial. Sci Rep. (2016) 6:31838. 10.1038/srep31838 27554803 PMC4995486

[27] Storey RF Angiolillo DJ Bonaca MP Thomas MR Judge HM Rollini F. Platelet inhibition with ticagrelor 60 mg versus 90 mg twice daily in the PEGASUS-TIMI 54 trial. J Am Coll Cardiol. (2016) 67:1145–54. 10.1016/j.jacc.2015.12.062 26965534

[28] Li J Qiu H Yan L Guo T Wang Y Li Y. Efficacy and safety of ticagrelor and clopidogrel in East Asian patients with coronary artery disease undergoing percutaneous coronary intervention. Curr Med Res Opin. (2020) 36:1739–45. 10.1080/03007995.2020.1825364 32945695

[29] Hjortbak MV Olesen KKW Seefeldt JM Lassen TR Jensen RV Perkins A. Translation of experimental cardioprotective capability of P2Y(12) inhibitors into clinical outcome in patients with ST-elevation myocardial infarction. Basic Res Cardiol. (2021) 116:36. 10.1007/s00395-021-00870-y 34037861

[30] Raphael Lieder H Tsoumani M Andreadou I Schrör K Heusch G Kleinbongard P. Platelet-mediated transfer of cardioprotection by remote ischemic conditioning and its abrogation by aspirin but not by ticagrelor. Cardiovasc Drugs Ther. (2022). 10.1007/s10557-022-07345-9 [Epub ahead of print]. PMC10517043 35595877

[31] Seung H Wrobel J Wadle C Bühler T Chiang D Rettkowski J. P2Y(12)-dependent activation of hematopoietic stem and progenitor cells promotes emergency hematopoiesis after myocardial infarction. Basic Res Cardiol. (2022) 117:16. 10.1007/s00395-022-00927-6 35353230 PMC8967792

[32] Cuisset T Deharo P Quilici J Johnson TW Deffarges S Bassez C. Benefit of switching dual antiplatelet therapy after acute coronary syndrome: the TOPIC (timing of platelet inhibition after acute coronary syndrome) randomized study. Eur Heart J. (2017) 38:3070–8. 10.1093/eurheartj/ehx175 28510646

[33] Mehran R Baber U Sharma SK Cohen DJ Angiolillo DJ Briguori C. Ticagrelor with or without aspirin in high-risk patients after PCI. N Engl J Med. (2019) 381:2032–42. 10.1056/NEJMoa1908419 31556978

[34] Kubica J Adamski P Gorog DA Kubica A Jilma B Budaj A. Low-dose ticagrelor with or without acetylsalicylic acid in patients with acute coronary syndrome: rationale and design of the ELECTRA-SIRIO 2 trial. Cardiol J. (2022) 29:148–53. 10.5603/CJ.a2021.0118 34622433 PMC8890404

